# Accuracy of high-sensitive troponin depending on renal function for clinical outcome prediction in patients with acute heart failure

**DOI:** 10.1007/s00380-021-01890-3

**Published:** 2021-06-21

**Authors:** Jakob Ledwoch, Anna Krauth, Jana Kraxenberger, Alisa Schneider, Katharina Leidgschwendner, Vera Schneider, Alexander Müller, Karl-Ludwig Laugwitz, Christian Kupatt, Eimo Martens

**Affiliations:** 1 Klinik Und Poliklinik Für Innere Medizin I, Klinikum Rechts Der Isar, Technical University of Munich, Munich, Germany; 2 Klinik Für Kardiologie, Pneumologie Und Internistische Intensivmedizin, München Klinik Neuperlach, Munich, Germany; 3 DZHK (German Center for Cardiovascular Research), Partner Site Munich Heart Alliance, Munich, Germany

**Keywords:** Mortality prediction, Acute heart failure, Troponin, Renal failure

## Abstract

High-sensitive troponin T (hs-TnT) is increasingly used for clinical outcome prediction in patients with acute heart failure (AHF). However, there is an ongoing debate regarding the potential impact of renal function on the prognostic accuracy of hs-TnT in this setting. The aim of the present study was to assess the prognostic value of hs-TnT within 6 h of admission for the prediction of 30-day mortality depending on renal function in patients with AHF. Patients admitted to our institution due to AHF were retrospectively included. Clinical information was gathered from electronic and paper-based patient charts. Patients with myocardial infarction were excluded. A total of 971 patients were enrolled in the present study. A negative correlation between estimated glomerular filtration rate (eGFR) and hsTnT was identified (Pearson *r* = − 0.16; *p* < 0.001) and eGFR was the only variable to be independently associated with hsTnT. The area under the curve (AUC) of hs-TnT for the prediction of 30-mortality was significantly higher in patients with an eGFR ≥ 45 ml/min (AUC 0.74) compared to those with an eGFR < 45 ml/min (AUC 0.63; *p* = 0.049). Sensitivity and specificity of the Youden Index derived optimal cut-off for hs-TnT was higher in patients with an eGFR ≥ 45 ml/min (40 ng/l: sensitivity 73%, specificity 71%) compared to patients with an eGFR < 45 ml/min (55 ng/l: sensitivity 63%, specificity 62%). Prognostic accuracy of hs-TnT in patients hospitalized for AHF regarding 30-day mortality is significantly lower in patients with reduced renal function.

## Introduction

One of the main pathological processes in acute heart failure (AHF) is myocardial injury, which can be detected by increased levels of cardiac troponins. In the setting of AHF, troponin is not only used to detect myocardial injury but can also be helpful for prognostication. A number of studies have found an association of elevated troponin measured by conventional assays with both in-hospital and long-term mortality [1–4]. The prognostic potential of troponin was confirmed also after the introduction of high-sensitive assays [5]. However, elevated high-sensitive troponin above the 99th percentile upper reference limit (URL) is very common in patients hospitalized with AHF. In a sub-analysis of the RELAX-AHF study assessing 1074 AHF patients, abnormal high-sensitive troponin T (hs-TnT) values were found in 90% of the study population. Furthermore, AHF patients often have concomitant renal dysfunction, which is also known to be associated with increased hs-TnT levels [6, 7]. One can expect that the coincidence of AHF and renal dysfunction almost always leads to elevated hs-TnT levels, which would limit its utility in this subset of patients. Studies analyzing troponin for the diagnosis of myocardial infarction already demonstrated that adjustment of hs-TnT cut-offs improves diagnostic accuracy in patients with renal failure [7, 8]. Since hs-TnT is also considered to be used for triage and outcome prediction in patients with AHF [9, 10] renal function should be taken into account when using this parameter in this setting. However, no data are available assessing hs-TnT for clinical outcome prediction in AHF depending on renal function. The present study sought to evaluate hs-TnT based risk prediction in patients with different classes of renal function admitted for AHF.

## Methods

### Study cohort

Patients aged ≥ 18 years presenting with AHF in our institution were included in a single-center retrospective AHF registry. Participants were enrolled between 2012, the year when hs-TnT was implemented into clinical routine in our institution, and 2019. Patient informed consent was waived due to the retrospective nature of the study. The study was approved by the hospital’s ethics committee and performed according to the Declaration of Helsinki.

AHF was diagnosed according to current guidelines [11]. It included the new onset of HF and acute decompensation of chronic HF. For the present analysis, exclusion criteria used were missing hs-TnT and creatinine measurements, current need for dialysis, patients with respiratory failure (defined as the need for mechanical ventilation), cardiogenic shock (defined as systolic blood pressure < 90 mm Hg or need for catecholamine therapy to maintain a systolic pressure ≥ 90 mm Hg together with clinical signs of impaired end-organ perfusion) and type 1 myocardial infarction, which was diagnosed according to the fourth universal definition of myocardial infarction [12]: Rise and/or fall of hs-TnT with at least one value above the 99th percentile URL and with at least one of the following: (I) symptoms suggestive for the acute coronary syndrome (chest pain or dyspnea); (II) new ischemic ECG changes (ST-segment depression, T-wave inversion, new pathological Q waves); (III) new wall motion abnormalities consistent with ischemic aetiology and (IV) identification of coronary thrombus by invasive angiography. Patients with the rise and/or fall of hs-TnT with at least one value above the 99th percentile URL and clinical symptoms (chest pain or dyspnea) but without other criteria for myocardial infarction (new ischemic ECG changes, new wall motion abnormalities consistent with ischemic aetiology or identification of coronary thrombus by invasive angiography) were classified as not having type 1 myocardial infarction [12]. Clinical information including the diagnosis of AHF and myocardial infarction was reviewed independently by two adjudicators (J.L. and A.S.).

### Patient data assessment

Patient data were extracted from electronic charts and paper-based document files. In each patient information regarding medical history, clinical signs and symptoms on hospital admission, ECG results, echocardiographic examinations and laboratory measurements were obtained. Clinical outcome was assessed until 30 days following hospital admission.

### Laboratory measurements and endpoint definition

Only patients with at least two hs-TnT measurements within 6 h following hospital admission were included to assess dynamic changes of hs-TnT and distinguish AHF from and myocardial infarction. Concentration of hs-TnT was analyzed by Elecsys hs-TnT assay (Roche Diagnostics, Basel, Switzerland). Renal function was quantified by an estimated glomerular filtration rate (eGFR) using the Chronic Kidney Disease Epidemiology Collaboration formula [13] based on plasma creatinine levels, age and gender. Patients were categorized into different groups of renal function according to KDIGO 2012 CKD guidelines [14].

Primary outcome measure was the assessment of the predictive value of maximum hs-TnT within 6 h of hospital admission with respect to 30-day mortality depending on renal function.

### Statistical analysis

Continuous variables were tested for normal distribution by Kolmogorov–Smirnov using Lilliefors correction and reported as median with interquartile range (IQR) or means with standard deviation. Categorical variables were expressed as numbers and percentages. Between-group comparisons across the different groups of renal function were performed using Kruskal–Wallis test or ANOVA for continuous data and Chi-Square or Fisher’s exact test for categorical data. A correlation analysis was performed between eGFR and hs-TnT using the Pearson correlation coefficient. To identify variables independently associated with hs-TnT a linear regression model was built. Variables were entered into this model in case they showed statistical significance in univariate analysis. The variables included were eGFR, age, arterial hypertension, dyslipidemia, smoker, diabetes mellitus, coronary artery disease, peripheral edema, diastolic blood pressure and heart rate. Furthermore, a number or receiver operating characteristic (ROC) analyses were conducted to assess the area under the curve (AUC) for maximum hs-TnT within 6 h of admission in predicting 30-day mortality in different categories of renal function. AUC comparison was performed using the *z* test. The prediction analysis included the calculation of a ROC derived hs-TnT cut-off using the Youden index defined by the minimal distance of the ROC curve to the point (0;1) of the graph. Hypothesis testing was two-tailed and a *p* value < 0.05 was considered as significant. Statistical analyses were performed using SPSS, version 26 (IBM, Chicago, USA).

## Results

### Study population and baseline characteristics

**Fig. 1:**
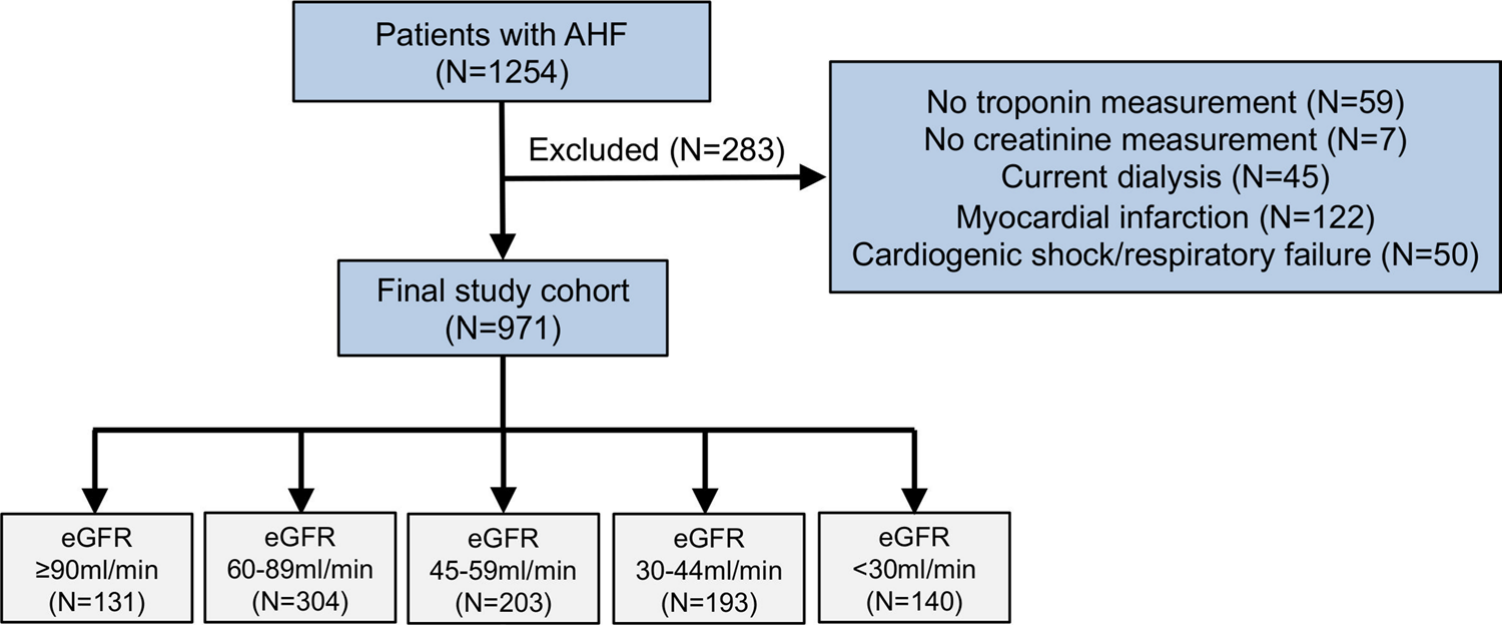
Study flow chart. *AHF* Acute heart failure, *eGFR* Estimated glomerular filtration rate

A total of 1254 patients were included in the registry. After the exclusion of 283 patients, 971 patients were available for the current analysis. Of them, 131 patients had eGFR ≥ 90 ml/min, 304 patients had eGFR 60–89 ml/min, 203 patients had eGFR 45–59 ml/min, 193 patients had eGFR 30–44 ml/min and 140 patients had eGFR < 30 ml/min. The study flow is illustrated in Fig. 1.

Baseline characteristics across the different classes of renal function are presented in Table 1. Patients with declining renal function were older, had more often diabetes mellitus and they had more rather more often coronary artery disease. Clinical signs and symptoms between the groups were similar except for peripheral edema showing higher prevalence in patients with declining renal function. With respect to echocardiographic data no differences were found between the groups.

**Table 1:** Baseline characteristics

	eGFR (ml/min)					*p* value
	≥ 90 (*N* = 131)	60–89 (*N* = 304)	45–59 (*N* = 203)	30–44 (*N* = 193)	< 30 (*N* = 140)	
Age (years)	68 (60–79)	74 (54–81)	77 (64–81)	79 (77–87)	81 (71–83)	< 0.001
Female	34% (45)	45% (137)	41% (83)	52% (100)	49% (68)	0.02
Arterial hypertension	81% (106)	82% (250)	93% (188)	87% (167)	86% (121)	0.009
Dyslipidemia	45% (59)	38% (114)	51% (103)	45% (86)	53% (74)	0.01
Smoker	48% (63)	38% (115)	31% (62)	30% (58)	31% (44)	0.004
Diabetes mellitus	29% (38)	26% (78)	33% (67)	43% (82)	49% (68)	< 0.001
Coronary artery disease	53% (70)	42% (128)	57% (115)	51% (99)	58% (81)	0.004
Atrial fibrillation	60% (79)	68% (206)	66% (133)	66% (128)	67% 94)	0.66
Clinical signs and symptoms						
Pulmonary congestion	70% (92)	79% (239)	82% (166)	75% (145)	79% (110)	0.14
Peripheral edema	74% (97)	67% (203)	77% (157)	77% (148)	81% (114)	0.007
NYHA ≥ III	95% (124)	93% (283)	95% (193)	91% (176)	91% (127)	0.41
Vital signs						
Oxygen saturation (%)	96 (88–97)	95 (93–96)	95 (89–97)	93 (88–97)	89 (88–96)	0.27
Systolic blood pressure (mmHg)	140 (126–151)	145 (126–165)	140 (111–166)	131 (123–143)	148 (132–158)	0.12
Diastolic blood pressure (mmHg)	96 (70–100)	90 (80–100)	75 (68–90)	80 (71–87)	87 (77–107)	< 0.001
Heart rate (beats/min)	87 (77–107)	95 (71–107)	93 (67–106)	80 (73–98)	75 (62–84)	< 0.001
Echocardiographic results						
LV-EF (%)	50 (40–60)	43 (30–50)	40 (30–50)	40 (32–50)	45 (38–55)	0.94
LV-EF ≥ 50%	43% (47)	44% (113)	43% (74)	41% (66)	46% (54)	0.94
LV-EF 40–49%	23% (25)	23% (58)	31% (37)	24% (39)	23% (27)	0.98
LV-EF < 40%	34% (38)	34% (87)	36% (63)	35% (56)	31% (36)	0.91
LVEDD (mm)	50 (46–58)	51 (45–60)	54 (48–63)	47 (42–57)	46 (43–55)	0.06
LVESD (mm)	38 (32–42)	39 (36–47)	43 (35–56)	38 (31–46)	35 (31–42)	0.09
TAPSE (mm)	18 (15–22)	17 (15–22)	15 (14–17)	15 (12–18)	18 (14–20)	0.74
RV-RA gradient (mmHg)	40 (32–40)	40 (31–47)	40 (33–40)	40 (34–48)	50 (40–58)	0.59
Laboratory findings						
Creatinine (mg/dl)	0.8 (0.7–0-9)	1.0 (0.8–1.2)	1.4 (1.2–1.4)	1.6 (1.5–1.7)	1.9 (1.8–2.6)	< 0.001
eGFR (ml/min)	116 (97–153)	70 (66–76)	50 (48–54)	37 (33–39)	26 (23–28)	< 0.001
Maximum hs-TnT (ng/l) within 6 h of admission	21 (15–35)	32 (18–48)	30 (28–39)	37 (30–49)	56 (40–81)	< 0.001
Maximum hs-TnT > 99th percentile URL (14 ng/l)	78% (102)	80% (242)	89% (180)	95% (184)	99% (138)	< 0.001

*eGFR* Estimated glomerular filtration rate, *LV-EF* Left ventricular ejection fraction, *LVEDD* Left ventricular enddiastolic diameter, *LVESD* Left ventricular endsystolic diameter, *TAPSE* Tricuspid annular plane systolic excursion, *RV-RA* Right ventricular right atrial, *Hs-TnT* High-sensitive troponin T, *URL* upper reference limit

### Association of renal function with hs-TnT levels

**Fig. 2:**
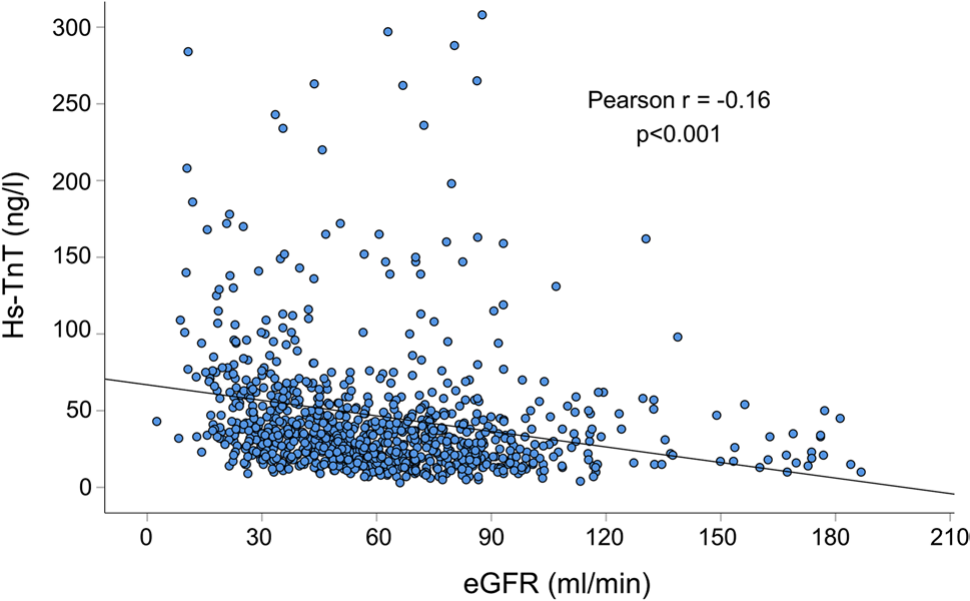
Correlation of hs-TnT with eGFR. *Hs-TnT* High sensitive troponin T, *eGFR* Estimated glomerular filtration rate

Laboratory findings in the two groups are listed in Table 1. Here, indices for renal function and cardiac troponin were significantly higher in patients with lower eGFR. Increased hs-TnT above the 99th percentile URL (> 14 ng/l) for myocardial infarction was found in 78% of patients with eGFR ≥ 90 ml/min and in 99% of patients with eGFR < 30 ml/min. Figure 2 illustrates the correlation of hs-TnT with eGFR. A moderate negative correlation between the two parameters was found (Pearson *r* = − 0.16; *p* < 0.001). In the linear regression model only eGFR was identified to be independently associated with hs-TnT (Table 2).

**Table 2:** Independent association with maximum hs-TnT

	Standardized regression coefficient ß	*p* value
eGFR	− 0.17	< 0.001
Age	0.06	0.17
Arterial hypertension	− 0.06	0.19
Dyslipidemia	− 0.06	0.15
Smoker	− 0.03	0.56
Diabetes mellitus	0.01	0.87
Coronary artery disease	0.07	0.10
Peripheral edema	− 0.04	0.33
Diastolic blood pressure	− 0.02	0.60
Heart rate	− 0.06	0.15

*eGFR* Estimated glomerular filtration rate

### Prognostic performance of hs-TnT cut-offs regarding 30-day mortality depending on renal failure

**Fig. 3:**
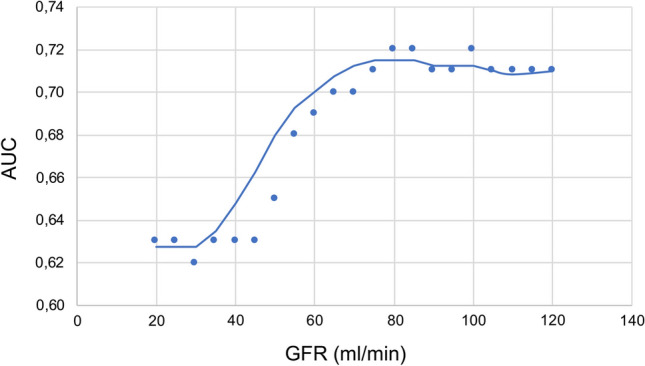
Area under the curve for hs-TnT regarding the prediction of 30-day mortality depending on renal function. *AUC* Area under the curve, *eGFR* Estimated glomerular filtration rate

In the present study 30-day mortality was 9.6% (*n* = 93) without a significant association with eGFR [OR 1.002 (95% CI 0.996–1.007); *p* = 0.51]. A scatter plot of the AUC of maximum hs-TnT within 6 h after hospital admission for the prediction of 30-day mortality depending on different levels of renal function is displayed in Fig. 3. It shows the AUC being stable between 0.72 and 0.70 until an eGFR of 60 ml/min. Between an eGFR of 60 ml/min and 45 ml/min a marked decline of the AUC was observed. Below an eGFR of 45 ml/min the AUC was stable again at values of 0.63. Similar to Fig. 3, and Table 3 depicts the AUC of hs-TnT depending on various classes of renal function. It demonstrates comparable levels of AUC in several stages of the renal function above an eGFR of 45 ml/min. The AUC clearly decreased below an eGFR of 45 ml/min.

**Table 3:** Area under the curve for hs-TnT regarding the prediction of 30-day mortality in different classes of renal function

eGFR	*n*	ROC AUC (95% CI)
≥ 90 ml/min	131	0.75 (0.64–0.86)
60–89 ml/min	304	0.74 (0.62–0.85)
45–59 ml/min	203	0.77 (0.64–0.91)
30–44 ml/min	193	0.63 (0.50–0.75)
< 30 ml/min	140	0.62 (0.49–0.74)

*eGFR* Estimated glomerular filtration rate, *ROC* Receiver operating characteristic, *AUC* Area under the curve

**Fig. 4:**
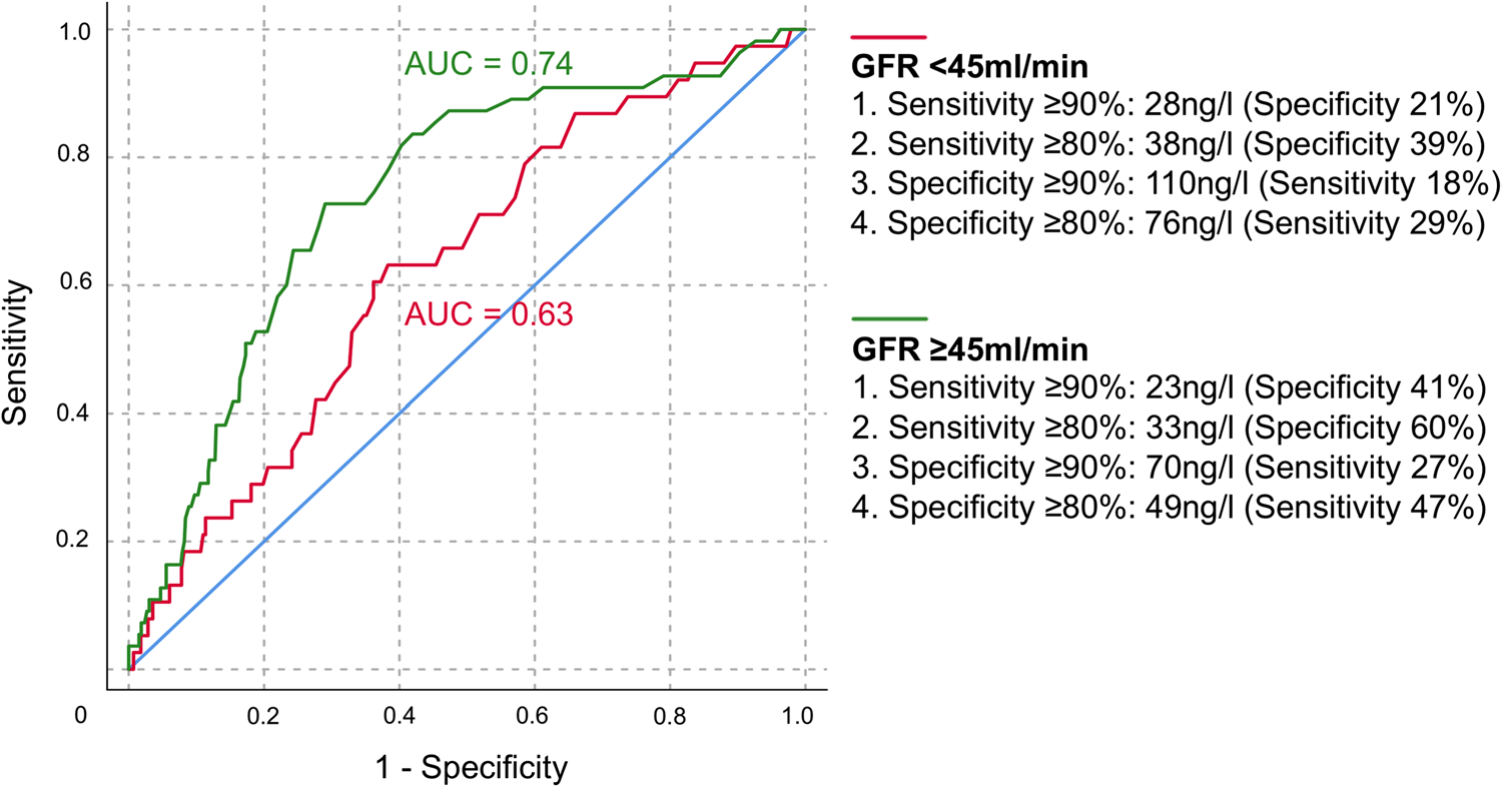
Predictive value of hs-TnT in patients with eGFR ≥ 45 ml/min versus < 45 ml/min regarding 30-day mortality. The difference between the AUC curves regarding hs-TnT in patients with eGFR ≥ 45 ml/min versus < 45 ml/min was significant (*p* = 0.049). *AUC* Area under the curve, *eGFR* Estimated glomerular filtration rate

A ROC analysis of hs-TnT predicting 30-day mortality in the group with eGFR ≥ 45 ml/min (AUC = 0.74) versus < 45 ml/min (AUC = 0.63) is displayed in Fig. 4. The difference in the AUC between both groups was significant [AUC difference 0.11 (95% CI 0.01–0.22); *p* = 0.049]. The AUC comparison of all other renal function groups regarding 30-day mortality did not show a statistically significant difference. Furthermore, hs-TnT values were presented depending on different cut-offs for sensitivity and specificity regarding the prediction of 30-day mortality in Fig. 4. The results show that higher values of hs-TnT were necessary for the group with eGFR < 45 ml/min to reach the same level of sensitivity and specificity, respectively. Additionally, the corresponding levels of sensitivity and specificity were lower in the group with eGFR < 45 ml/min (e.g. the corresponding specificity of and sensitivity ≥ 90% was 41% in the group with eGFR ≥ 45 ml/min compared to 21% in the group with eGFR < 45 ml/min). Youden Index optimized hs-TnT cut-offs depending on renal function for the prediction of 30-day mortality compared to the 99th percentile URL cut-off are presented in Table 4. An equation using renal function corrected hsTnT levels (hsTnT × eGFR) did not improve diagnostic accuracy in these patients with eGFR < 45 ml/min (AUC 0.62).

**Table 4:** Youden Index optimized hs-TnT cut-offs depending on renal function

	hs-TnT cut-off	Sensitivity	Specificity
eGFR ≥ 45 ml/min	40 ng/l	73%	71%
	99th percentile URL (14 ng/l)	93%	13%
eGFR < 45 ml/min	55 ng/l	63%	62%
	99th percentile URL (14 ng/l)	100%	2%

*eGFR* Estimated glomerular filtration rate, *ROC* Receiver operating characteristic, *AUC* Area under the curve

## Discussion

The present study is the first assessing the diagnostic value of renal function-dependent hs-TnT cut-offs for clinical outcome prediction in patients presenting with AHF. The main results are that (I) higher hs-TnT cut-off values than the 99th percentile URL used for myocardial infarction diagnosis are necessary for an adequate outcome prediction and that (II) outcome prediction using hs-TnT is less accurate in patients with eGFR < 45 ml/min in the setting of AHF.

### Association of hs-TnT levels with renal function

Elevated troponin levels are very common in patients with decreased renal function. A study assessing non-myocardial infarction related causes of increased troponin in emergency departments showed renal dysfunction to be responsible in more than half of the patients [15]. On the one hand, the association of increased hs‐TnT and kidney failure may be due to cardiac stress in the context of the cardiorenal syndrome [16]. On the other hand, reduced renal clearance of hs‐TnT or its fragments in patients with kidney failure may lead to elevated and chronic detectable levels of hs‐TnT [17, 18]. There is still uncertainty regarding the renal clearance of cardiac TnT since it has a molecular weight of 37 kDa, indicating a low rate of filtration through the glomerular membrane. However, circulating cardiac TnT measured by the Roche high-sensitive TnT assay is mostly degraded to < 20 kDa fragments showing that cardiac TnT can be partly cleared via the kidneys [19, 20]. Moreover, a recent PET study in rats showed that the liver and kidneys are responsible for cardiac troponin clearance [21]. This would support the hypothesis of increased hs‐TnT levels in those with reduced renal function. Furthermore, Friden et al. demonstrated that particularly only moderately elevated cardiac TnT levels as seen in heart failure patients show a relevant renal clearance [20].

It is also known that AHF is associated with an increase in cardiac troponin. This can be caused by different mechanisms including wall stress, altered calcium handling, endogenous catecholamines, oxidative stress and cytokines leading to cytosolic mobilization of troponin, apoptosis and/or cell necrosis [22]. Studies report the prevalence of elevated hs-TnT above the 99th percentile URL (> 14 ng/l) to vary between 77 and 90% in AHF [9, 23, 24]. Our study confirms these previous findings. Increased hs-TnT above the 99th percentile URL was found in 79% of patients with preserved renal function and reached a rate of 94% in those with renal failure (*p* < 0.001) in the setting of AHF. These numbers clearly demonstrate that almost all patients hospitalized with AHF and concomitant renal failure show elevated hs-TnT values. Therefore, using the conventional hs-TnT cut-off for myocardial infarction (99th percentile URL of 14 ng/l) is of limited value for any risk prediction in this subset of patients. Moreover, we were able to show a significant correlation of hs-TnT with eGFR. Increased hs-TnT values were observed with declining eGFR. Furthermore, in our linear regression model eGFR was the only variable to be independently associated with hs-TnT. This result underlines our initial hypothesis of a strong association of hs-TnT with eGFR and, hence, it indicates that the hs-TnT based clinical outcome prediction should be to be assessed dependent on renal function.

### Diagnostic value of troponin for the prediction of 30-day mortality

Since cardiac troponins are released from cardiomyocytes due to various clinical causes in various clinical settings (e.g. myocardial infarction, AHF, stroke, etc.), different levels of troponin are found in each of those clinical scenarios. As a consequence, not one specific cut-off can uniformly be applied as a diagnostic or prognostic measure in all these settings. Parissis et al. identified a ROC optimized hsTnT cut-off of 77 ng/l to predict all-cause mortality in AHF patients with a sensitivity of 62% and specificity of 72%. Roset et al. found a ROC optimized hsTnT cut-off of 35 ng/l with a sensitivity of 67% and specificity of 56%. The cut-off was lower in the later analysis because of less comorbidities in this patient cohort. The ROC optimized hs-TnT limits in the present work of 40 ng/l and 55 ng/l depending on renal function are in line with those previous reports and indicate which range of hs-TnT measures should be anticipated to be of prognostic significance in AHF. The recently published High Sensitivity Troponin T Rules Out Acute Cardiac Insufficiency Trial (TACIT) failed to identify patients at low risk presenting with AHF in the ED [9]. However, the 99th percentile URL of hs-TnT (14 ng/l) was used as triage cut-off in this trial, which is relatively low. Furthermore, 26% of the patients had chronic renal failure, which most probably contributed to this negative study result. In contrast, in a sub-analysis from this study a higher hs-TnT cut-off of 19 ng/l was significantly associated with short-term mortality, which indicate that higher hs-TnT values are necessary to increase prognostic accuracy in the setting of AHF. The present results showed that the 99th percentile URL of 14 ng/l did not provide sufficient diagnostic accuracy due to very low specificity.

Importantly, our findings demonstrate the optimal cut-off of hs-TnT for clinical outcome prediction in AHF not only to be generally higher than the 99th percentile URL but also to be highly dependent on renal function. The same observation was made in studies analyzing hs-TnT cut-offs in the setting of myocardial infarction and renal failure. In patients with renal failure a hs-TnT cut-off for diagnosing myocardial infarction was twofold to threefold higher than in those without renal failure [7, 8]. In the present study, the ROC analysis in different stages of renal function showed a distinctively decreased AUC in patients with an eGFR < 45 ml/min. This finding facilitates the transition into clinical practice by pointing out a clear eGFR cut-off, which can be used to identify patients in whom hs-TnT provide inadequate diagnostic accuracy. In addition, a correction for renal function using an eGFR adjusted hs-TnT level did not improve diagnostic accuracy. Therefore, hs-TnT should be utilized with caution as a prognostic factor in patients with AHF showing an eGFR < 45 ml/min.

### Limitations

The work represents a retrospective single-center analysis and, hence, generalization of the results should be performed with caution. Since NT-pro-BNP was not routinely measured, combination with this parameter to enhance prognostication was not possible. No other assays of cardiac troponins were used and, therefore, only hs-TnT were assessed in this setting. Since the diagnosis of renal failure was based on a single estimate of renal function at admission, it is unclear whether acute kidney injury or chronic kidney disease was present. However, both entities are associated with increased cardiovascular risk and the used approach is consistent with a clinical practice where renal function and cardiac troponin are measured at the same time in the emergency department. Long-term outcome could not be assessed since no follow-up beyond 30 days was performed.

## Conclusion

Hs-TnT shows adequate prognostic accuracy for the identification of 30-day mortality in patients with eGFR ≥ 45 ml/min hospitalized for AHF in contrast to those with an eGFR < 30 ml/min. Whether the use of AHF adjusted hs-TnT thresholds can be implemented for prognostication in patients without severely decreased eGFR in clinical practice needs to be confirmed in future large prospective trials.
